# Obesity-Hypoventilation Syndrome: Increased Risk of Death over Sleep Apnea Syndrome

**DOI:** 10.1371/journal.pone.0117808

**Published:** 2015-02-11

**Authors:** Olalla Castro-Añón, Luis A. Pérez de Llano, Sandra De la Fuente Sánchez, Rafael Golpe, Lidia Méndez Marote, Julián Castro-Castro, Arturo González Quintela

**Affiliations:** 1 Respiratory Division and Sleep Disorders Unit, Lucus Augusti University Hospital, Galician Health Service, Lugo, Spain; 2 Lucus Augusti University Hospital, Galician Health Service, Lugo, Spain; 3 Galician Health Service, Lugo, Spain; 4 Department of Medicine, University of Santiago de Compostela, Santiago de Compostela, Spain; Weill Cornell Medical College Qatar, QATAR

## Abstract

**Aim:**

To study whether mortality and cardiovascular morbidity differ in non-invasive ventilation (NIV)-treated patients with severe obesity-hypoventilation syndrome (OHS) as compared with CPAP-treated patients with obstructive sleep apnea syndrome (OSAS), and to identify independent predictors of mortality in OHS.

**Material and methods:**

Two retrospective cohorts of OHS and OSAS were matched 1:2 according to sex, age (±10 year) and length of time since initiation of CPAP/NIV therapy (±6 months).

**Results:**

Three hundred and thirty subjects (110 patients with OHS and 220 patients with OSAS) were studied. Mean follow-up time was 7±4 years. The five year mortality rates were 15.5% in OHS cohort and 4.5% in OSAS cohort (p< 0.05). Patients with OHS had a 2-fold increase (OR 2; 95% CI: 1.11–3.60) in the risk of mortality and 1.86 fold (OR 1.86; 95% CI: 1.14–3.04) increased risk of having a cardiovascular event. Diabetes, baseline diurnal SaO_2_ < 83%, EPAP < 7 cmH2O after titration and adherence to NIV < 4 hours independently predicted mortality in OHS.

**Conclusion:**

Mortality of severe OHS is high and substantially worse than that of OSAS. Severe OHS should be considered a systemic disease that encompasses respiratory, metabolic and cardiovascular components that require a multimodal therapeutic approach.

## Introduction

Obesity hypoventilation syndrome (OHS) is a clinical entity characterized by the coexistence of obesity and hypercapnia during wakefulness [1–3]. However, the lack of a standardized definition of OHS in general, and of OHS—obstructive sleep apnea syndrome (OSAS) relationships in particular, leads to confusion [4, 5]. It is fundamental to distinguish between OHS and OSAS since their natural history and clinical course will diverge and therapy is conceptually different (continuous positive airway pressure—CPAP- is the cornerstone of treatment in OSAS and non invasive ventilation—NIV- is essential in OHS) [6]. Although the prevalence of OHS remains largely unknown, it is currently estimated at 0.15–0.3% in the US population [7]. On the contrary, it was reported that 2% of women and 4% of men have OSAS [8].

Priou et al, [9] in a cohort of 130 patients with NIV-treated OHS, found a five-year 77.3% probability of survival. On the other hand, Campos Rodriguez et al observed a five-year 85.5% cumulative survival rate in their series of 871 CPAP-treated OSAS patients [10]. In view of these results, it could be concluded that mortality is higher in OHS than in OSAS. However, an outcome direct-comparison between these two entities is lacking.

The aims of this study were (1) to verify whether mortality differs in NIV-treated patients with severe OHS as compared with CPAP-treated patients with OSAS, (2) to evaluate whether cardiovascular morbidity is different between these two groups, and (3) to identify the independent predictors of mortality in a cohort of NIV-treated OHS subjects.

## Material and Methods

### Design of the study

**Fig 1:**
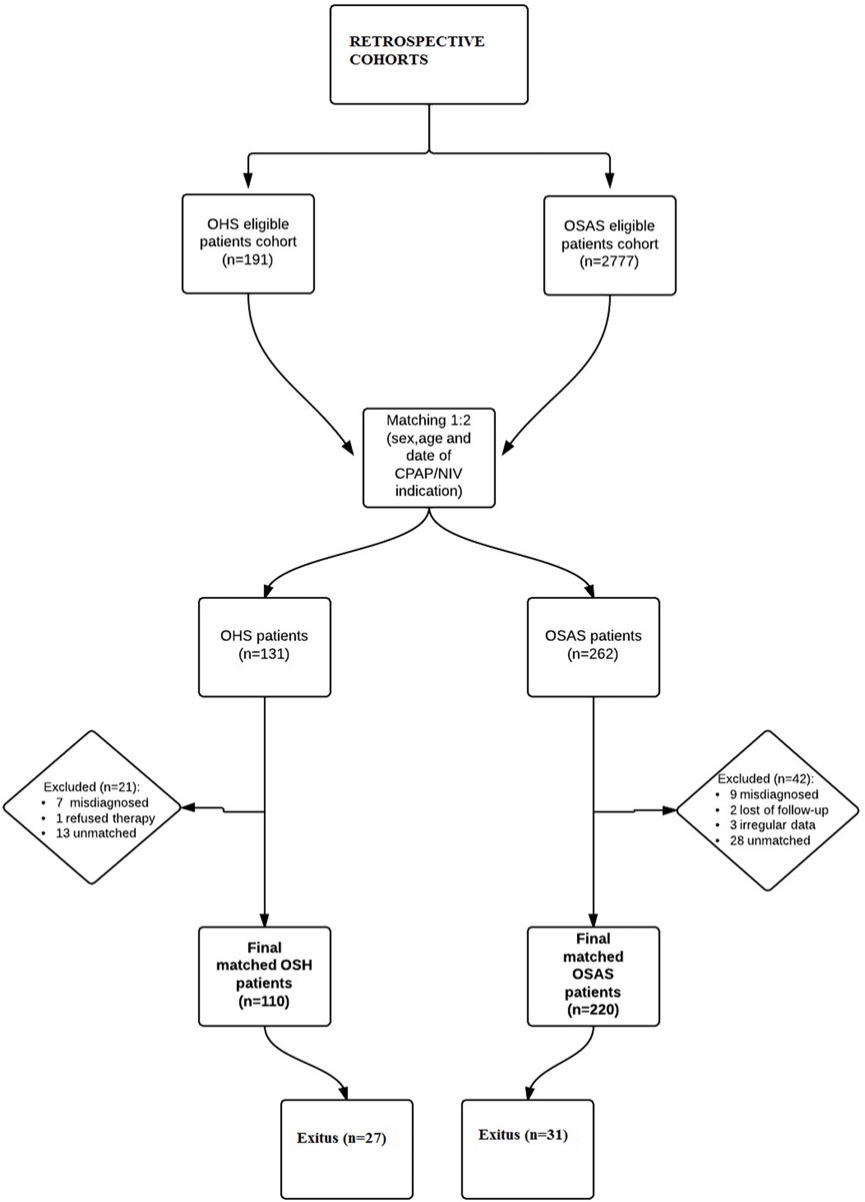
Flow-chart diagram of the study.

This retrospective matched cohort investigation included study and comparison cohorts from a single 823 bed university hospital. We first selected the study cohort by identifying those subjects who had been diagnosed of severe OHS (hypercapnic respiratory failure at the time of diagnosis) from 1995 to 2010 (191 patients). Then, we selected the comparison cohort: 2777 subjects diagnosed with OSAS during the same period. The groups of OHS and OSAS were individually matched 1:2 according to sex, age (±10 year) and length of time since initiation of CPAP/NIV therapy (±6 months) to reduce bias from the confounding effects of these variables (sex and age can influence mortality in patients with sleep-disordered breathing) [11]. A multivariate minimum-distance matching algorithm was used for computer selection of each set. The follow-up time was calculated in years from diagnosis to death or the last observation time (May 9, 2013). Finally, 110 OHS patients and 220 OSAS patients were included. The flow chart of the process is shown in Fig. 1.

Approval was obtained from the Ethical Committee of Galicia (Reg. Nº 2011/335). Patient records and information were anonymized and de-identified prior to analysis.

### Patients

Patients with severe OHS met all of the following criteria at the time of diagnosis: (1) obesity with a BMI of > 30 kg/m^2^; (2) hypercapnic respiratory failure (PaCO_2_ ≥ 6.7 and PaO_2_ < 8.0 kPa); (3) FEV1/FVC ratio of ≥ 70%; (4) the absence of any respiratory disorder that could account for gas-exchange disturbances (eg, kyphoscoliosis or diaphragmatic paralysis). We decided to exclude patients with mild hypercapnia to be sure that we were dealing with “real pickwickian patients”. We and other authors believe that there is a wide range of severity among patients with OHS, so prognosis and response to therapy may vary between individuals [6, 12]. By deciding to employ a restrictive definition of OHS, we wanted to select a homogeneous sample of OHS patients. Patients were diagnosed with OHS in a stable clinical state or during an exacerbation. Exacerbation of OHS was defined as respiratory difficulty that required urgent medical attention.

According to the Spanish guidelines [13], patients with apnea-hypopnea index ≥ 5 associated with excessive diurnal somnolence were considered to have OSAS. We excluded patients with a diagnosis of chronic obstructive pulmonary disease in the group of OSAS.

A patient diagnosed with OHS could also meet the definition of OSAS (in fact, that was the case in most of them: 87.9%), but the reverse was not possible. It was mandatory to have a global respiratory failure to be included in OHS group and only patients without hypoventilation could be chosen to be part of OSAS group.

Patients were excluded if they were aged < 18, if they were lost to follow-up, if they were not possible to match, if they refused CPAP or NIV, and if we had incomplete data.

### Measurements

Baseline demographic and clinical characteristics and follow-up outcomes were recorded. Comorbidities identified at the time of diagnosis included arterial hypertension, diabetes mellitus, medical history of congestive heart failure or arrhythmia and clinical manifestations of atherosclerosis, including coronary artery disease, cerebrovascular disease and peripheral arterial disease. All cardiovascular and respiratory events during follow-up were extracted from patient charts. Under the term “serious cardiovascular events”, cardiac ischemic episodes, cardiac arrhythmia, stroke, leg, bowel or kidney ischemia, were comprehended. Causes of death were collected from medical records.

At the time of diagnosis, all patients underwent a clinical examination, a spirometry and a sleep study. Spirometry was performed using Sibelmed Datospir 120 spirometer (Sibel S.A, Barcelona, Spain), according to the ATS/ERS consensus [14]. The predicted values used were those of the European Respiratory Society [15]. Arterial blood gas analysis was obtained in every OHS patient but only exceptionally in OSAS patients, since pulse-oximetry-measured arterial blood saturation was usually normal in them. A fasting early morning sample was obtained, with the patient in a sitting position and on room air.

OSAS was diagnosed either by full night polysomnography (ALICE 3 and ALICE 5, Respironics, Murrysville, PA; XLTEC-Connex polysomnography system, Oakville, ON, Canada) or by all-night attended cardiorespiratory polygraphy (BREAS SC-20, Breas Medical S.L., Mölnlyke, Sweden; Embletta pds; ResMed, Spain). Sleep and respiratory events were defined according to the classical Rechtschaffen and Kales criteria [16] and according to the Spanish guidelines [13].

Transcutaneous oxygen saturation (SaO_2_) was measured with a pulse oximeter (Pulsox 3i; Minolta, Ramsey NJ). The mean nighttime SaO_2_ (mnSaO_2_), percentage of recording time with SaO_2_ <90% (CT90%) and the nocturnal desaturation index (DI4%), defined as the number of dips in SaO_2_ ≥4% per hour of recording time, were calculated using computer software (Pulsox SaO_2_ analysis software DS-3; Minolta, Ramsey NJ).

The majority of OHS patients were initially treated with NIV as described elsewhere [17]. Some patients could be initially treated with CPAP if an acceptable level of nocturnal oxygen saturation (DI4% <10 and CT90% < 30%) was reached. During a second nighttime sleep study, the final NIV settings were established. Even if the patients were being treated with NIV, we first employed nasal continuous positive airway pressure (CPAP) in every case. The CPAP was adjusted with the aim of preventing apneas and hypopneas in all sleep stages and it was maintained if nocturnal oxygen saturation was above 90% most of the time. When significant oxygen desaturation persisted despite the use of high pressures, CPAP was considered to have failed, and we subsequently changed over to a pressure ventilator. Then, IPAP was increased until a maximum was reached and oxygen was added if required.

Empirical CPAP treatment was started in all patients who met OSAS definition and the effective therapeutic pressure was determined by manual or automated titration (Autoset T, S8 and S9, RESMED Co, Australia).

Objective adherence with treatment was assessed by the use of the built-in time counter on the device. Patients were considered as adherent when using their machine for more than 4 h per night for 70% of the observed nights [18].

### Statistical analysis

Outcome measures and analyses were established a priori according to standard operating procedures of the study [19]. Analyses were performed with SPSS Statistics version 17.0 software (SPSS, Inc., Chicago, Illinois), R version v2.12.0 freeware (R Development Core Team, R Foundation for Statistical Computing), Microsoft Excel 2007 software (Microsoft Corp, Redmond, Was) and Visual Basic. Although we did not perform a power calculation a priori, a two-tailed post-hoc power calculation based on our main outcome variable (5 year survival rates in OHS and OSAS patients) revealed that the data set was large enough to obtain 88.7% power with α < 0.05. Statistically significant results were defined as P value of ≤ 0.05.

Descriptive statistics were used to characterize the patients. A Mantel—Haenszel chi-squared test was used to compare distributions of baseline categorical demographic and clinical parameters across the two cohorts. We handled categorization for some continuous variables according to the descriptive statistics, cluster analyses and clinical criteria. For continuous variables, means and distributions were compared between the matched sets by using univariate conditional logistic regression. In addition, multivariate conditional logistic regression was used to determine the prognostically independent variables associated with the two cohorts. Categorical variables are expressed as a percentage of the group of origin, while continuous variables are reported as mean ± standard deviation. Results for conditional logistic regression models are reported as odds ratios (OR) with 95% confidence interval (CI).

Survival analysis was performed using a weighted Kaplan-Meier estimator for matched data [20]. When multiple matching is handled, highly stratified data are produced, with strata containing possibly censored observations. For this reason, the usual nonparametric procedures, such as the log-rank test, cannot be directly addressed to compare survival in the two groups. The stratified version of these tests for matched data is inefficient when the number of strata increases and the stratum size is small [21]. Furthermore, these methods are less sensitive when proportional hazard is not satisfied.

At last, we focused on the OSH group. Univariate analyses of variables influencing overall survival (OS) were performed by log-rank test, which identified a preliminary list of significant factors. Variables with a significant association with OS in univariate analysis (*P*-value <0.05) were selected and evaluated by multivariate analysis. Multivariate analyses were performed by Cox proportional-hazards regression using the forward stepwise method.

## Results

Three hundred and thirty subjects (110 patients with OHS and 220 patients with OSAS; 43.6% women; mean age 64 ± 11 yr) were studied. Mean follow-up time was 7 ± 4 years (range: 1–15 years). Baseline data are shown in Tables 1 and 2.

**Table 1:** Demographic characteristics, cardiovascular risk factors and history of cardiovascular disease of the 2 cohorts.

	OHS group (n = 110)	OSAS group (n = 220)	Statistical difference.
**Age (yr)**	63.9 ± 11.1	63.4 ± 10.7	0.02
**Sex: M/F**	56.4% / 43.6%	56.4% / 43.6%	-
**Current smoker**	23.6%	20.0%	0.42
**Follow-up period (yr)**	6.8 ± 4.0	7.4 ± 3.8	< 0.001
**BMI (kg/m^2^)**	42.4 ± 8.0	34.9 ± 7.4	< 0.001
**Arterial hypertension**	74.5%	63.2%	0.028
**Dyslipidemia**	26.4%	41.8%	0.008
**Diabetes**	27.3%	24.1%	0.63
**Heart failure**	10.9%	4.1%	0.033
**Arrhythmia**	18.2%	10.0%	0.039
**Ischemic heart disease**	12.7%	13.2%	0.954
**Stroke**	2.7%	6.8%	0.185
**Systemic atherosclerosis**	3.6%	5.9%	0.536

Values are expressed as mean ± SD or No. of patients (%). BMI: body mass index.

**Table 2:** Epworth sleepiness scale, basal arterial blood gas results, sleep study findings and therapeutic intervention for the 2 cohorts.

	OHS group (n = 110)	OSAS group (n = 220)	Statistical difference.
**Epworth**	12.87± 5.95	13.02± 5.02	0.75
**PaO_2_ (kPa)**	6.57 ± 0.91	-	-
**PaCO_2_ (kPa)**	7.86 ± 1.26	-	-
**SaO_2_ (%)**	82.4 ± 7.8	94.9 ± 3.5	<0.01
**Presented with pH < 7.35**	26.6%	-	-
**AHI**	41.2 ± 27.6	46.2 ± 25.1	0.15
**DI4%**	43.1 ± 25.0	37.1 ± 25.5	0.06
**CT90%**	79.8 ± 23.4	28.3 ± 28.0	<0.01
**CPAP after titration (%)**	37.3%	98.2%	<0.01
**NIV after titration (%)**	60.9%	1.8%	<0.01
**Supplemental O_2_ after titration (%)**	52.3%	1.4%	<0.01
**CPAP after titration (cmH_2_0)**	9.2±2.4	8.9±1.7	0.32
**IPAP after titration (cmH_2_0)**	18.5±2.5		
**EPAP after titration (cmH_2_0)**	8.4±1.9		
**Adherence (hours/night).**	6.2 ± 3.0	5.8 ± 3.2	0.29

Values are expressed as mean ± SD or No. of patients (%). PaO2: arterial oxygen pressure; PaCO2: arterial carbon dioxide pressure; AHI: apnea-hypopnea index; DI4: nocturnal desaturation ≥ 4% index; CT90%: cumulative percentages of sleep time with SaO_2_ <90%; CPAP: continuous positive airway pressure; NIV: non invasive ventilation; IPAP: inspiratory positive airway pressure; EPAP: expiratory positive airway pressure.

Patients with OHS had significantly higher BMI than OSAS patients (42.4 vs 34.9 kg/m^2^; p < 0.001). A medical history of arterial hypertension (74% vs 63%; p = 0.028), heart failure (11% vs 4%; p < 0.001) and arrhythmia (18% vs 10%; p = 0.039) were more frequent in the OHS cohort. On the contrary, dyslipidemia was more frequent in OSAS patients (26% vs 42%; p = 0.008). We did not find significant differences between both cohorts in the frequency of diabetes, ischemic heart disease and stroke. Diurnal and nocturnal SaO2 were significantly lower in OHS patients but the severity of OSAS (defined by the AHI) was the same in both cohorts.

NIV therapy was started electively in 29.7% of OHS patients and following an episode of exacerbation in the remaining 70.3%. Approximately one quarter (26.6%) of the OHS sample presented with respiratory acidosis. Among OHS patients, 90% of them started with NIV and 10% with CPAP. After titration, mean IPAP was 18.5 ± 2.5 cmH2O, EPAP 8.4 ± 1.9 cmH2O, and 52% of the OHS population required supplemental oxygen. During follow-up, 11.3% patients discontinued NIV because of lack of acceptance, and 30.3% patients were switched to CPAP in the OHS cohort. CPAP treatment was withdrawn in 15.7% of the OSAS cohort patients due to non-compliance. No patient needed to switch from CPAP to NIV. The mean daily use at the most recent visit was 6.2 ± 3.0 h/d in patients continuing to use NIV, not significantly different from that observed in the OSAS cohort (5.8 ± 3.2 h/d).

**Fig 2:**
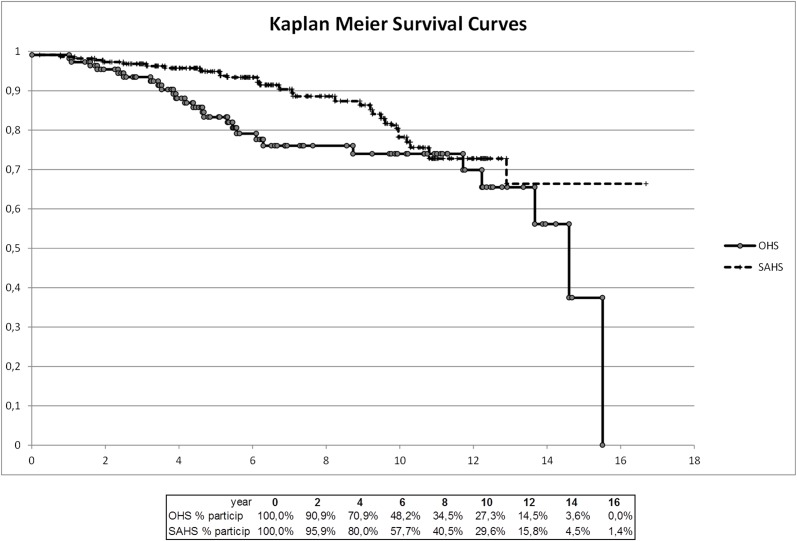
Kaplan-Meier survival curves for both groups.

**Fig 3:**
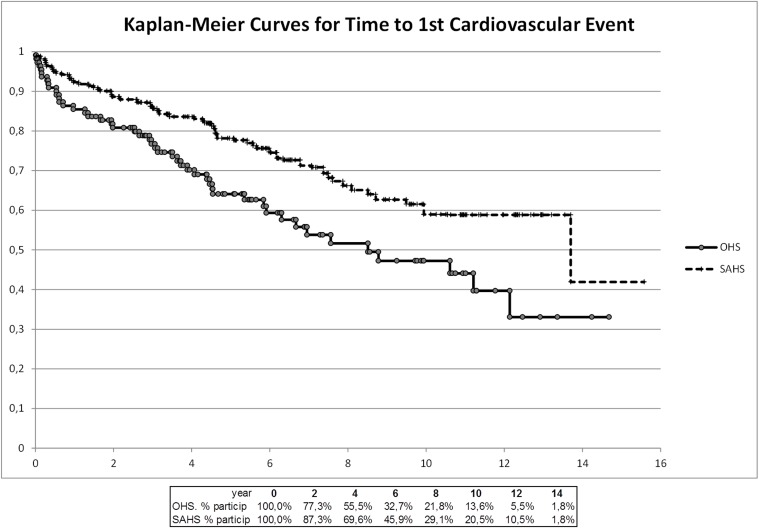
Kaplan Meier curves for time to first cardiovascular event.

During follow-up, 24.5% of patients with OHS and 14.1% of OSAS patients died (p < 0.05). The five year mortality rates were 15.5% and 4.5% respectively. Patients with OHS had a 2-fold increase (OR 2; 95% CI: 1.11–3.60) in the risk of mortality compared with those with OSAS. The main cause of death in the OHS cohort (48%) was cardiovascular diseases (heart failure 8 patients, sudden death 3 patients, ischemic heart disease 1 patient and stroke another 1). Other causes of death were cancer (15%), respiratory failure (11%), infections (11%), and others (15%). Cardiovascular diseases were also the most frequent cause of death in the OSAS cohort (48%). At least one serious cardiovascular event was diagnosed in 43.6% of the patients with OHS and 29.5% of OSAS patients during follow-up (p = 0.014). Patients with OHS were at 1.86 fold (OR 1.86; 95% CI: 1.14–3.04) increased risk of having a cardiovascular event compared with those with OSAS. Kaplan-Meier curves for survival and time to first cardiovascular events for the 2 cohorts are shown in Figs. 2 and 3.

Upon univariate analysis, medical history of diabetes, heart failure or arrhythmia, baseline diurnal SaO2 <83%, CPAP vs BiPAP at discharge, low levels of IPAP and EPAP at discharge and after titration, lack of adherence to NIV, switching from NIV to CPAP, and the occurrence of cardiorespiratory events during follow-up (particularly, episodes of heart failure) turned out to predict mortality in OHS patients (p < 0.05; Table 3). Interestingly, BMI, sleep respiratory variables and starting NIV in the acute clinical setting were not associated with a higher risk of death. When all significant prognostic factors in the univariate study were included in a multivariate analysis, the most powerful predictive factors were the occurrence of cardiovascular events in less than one year of follow up (HR 6.45; p = 0.029) and EPAP < 7 cmH2O after titration (HR 20,88; p = 0.003). However, when we discarded those variables linked to the patients’ outcome (such as cardiovascular events) and considered only anthropomorphic-, disease-, and treatment-related variables, clinical history of diabetes, baseline diurnal SaO2 < 83%, EPAP < 7 cmH2O after titration and adherence to NIV < 4 hours emerged as the most important variables to predict mortality (p < 0.05; Table 4).

**Table 3:** Univariate analysis of risk factors related to mortality in OHS patients.

	Hazard Ratio	95% CI	P value
**Diabetes vs no diabetes**	2.56	1.16–5.68	0.020
**History of heart failure vs no history of heart failure**	3.79	1.49–9.60	0.005
**History of arrhythmia vs no history of arrhythmia**	2.89	1.28–6.55	0.011
**Baseline SaO_2_ < 83% vs SaO_2_ ≥ 83%**	2.24	1.02–4.95	0.045
**CPAP vs BiPAP at discharge**	3.17	1.16–8.64	0.024
**EPAP < 7 vs EPAP ≥ 7 cmH_2_O at discharge**	3.74	1.42–9.86	0.008
**IPAP ≤ 17 vs IPAP > 17 cmH_2_O at discharge**	3.43	1.44–8.18	0.005
**BIPAP switched to CPAP vs no switched to CPAP after titration**	0.24	0.07–0.81	0.021
**EPAP < 7 vs EPAP ≥ 7cmH_2_O after titration**	3.20	1.03–9.97	0.045
**IPAP ≤ 17 vs IPAP > 17 cmH_2_O after titration**	3.03	1.13–8.12	0.027
**Adherence < 4 vs adherence ≥ 4 hours**	3.28	1.42–7.61	0.006
**CVE during follow-up vs no CVE**	8.40	2.51–28.15	<0.00001
**HF vs no HF**	6.40	2.56–16.02	<0.00001
**Cardiorespiratory events vs no cardiorespiratory events**	9.01	2.70–30.11	<0.00001
**Time to the first CVE < 1 year vs ≥ 1 year**	7.87	3.18–19.47	<0.00001

SaO2: oxygen saturation; NIV: non invasisve ventilation; CPAP: continuous positive airway pressure; EPAP: expiratory positive airway pressure; IPAP: inspiratory positive airway pressure; BIPAP: bilevel positive airway pressure; CVE: cardiovascular events; HF: heart failure.

**Table 4:** Independent predictors of mortality on Multivariate Analysis for OHS patients.

	Hazard Ratio	95% CI	*P* value
Diabetes vs no diabetes	5.28	1.51–18.50	0.009
Adherence < 4 vs adherence ≥ 4 hours	9.84	2.69–35.98	0.001
SaO_2_ < 83% vs SaO_2_ ≥ 83%	7.50	2.15–26.15	0.002
EPAP < 7 vs EPAP ≥ 7 cmH_2_O (after titration)	4.12	1.05–16.16	0.043

## Discussion

The first aim of this study was to confirm that NIV-treated OHS subjects were at increased mortality risk when compared to CPAP-treated OSAS individuals. OHS patients were found to be 2 times more likely to die. We constructed 2 matched cohorts to minimize confounding variables. However, several inevitable differences, implicit in the OHS definition, were observed: BMI was higher in the OHS cohort and significantly more OHS patients had a history of heart disease. It is therefore difficult to disentangle the impact of obesity from OHS itself. Nevertheless, it should be noted that, in univariate analysis, obesity was not significantly associated with mortality. We found that most of our patients died of heart failure. This is not surprising since it has been reported that patients with OHS are more likely to develop heart failure than obese individuals with eucapnia [22]. On the other hand, death from respiratory failure was infrequent in our experience, a fact that underlines the efficacy of NIV in this setting.

Previous studies showed that patients with OHS may experience higher morbidity and mortality than patients who are similarly obese and have OSAS [22, 23]. In a population of 276 individuals who consecutively underwent a PSG for suspected OSAS, OHS patients (38 of them) had a significantly higher prevalence of congestive heart failure than those with OSAS, but non-significantly higher rates of arterial hypertension, diabetes mellitus and hyperlipidemia [22]. The explanation for this probably relies on a different inflammatory status and endothelial function between OHS and eucapnic obese patients [24].

Our study showed a high mortality rate among OHS patients (15.5% at 5 years), even when they were correctly diagnosed and treated with NIV. However, our figures were lower to those reported by Priou et al (23% at 5 years) and remarkably lower to those published by Budweisser et al (30% at 5 years) [9, 25]. Mean BMI, adherence to NIV and mean inspiratory and expiratory pressures were similar in the 3 populations with the only disparity of a lower EPAP (about 6 cmH2O) in Budweisser’s study. Therefore, the reasons for the lower mortality rates in our study are unclear but may be related to differences in patient comorbidities or concomitant therapies. Interestingly, 52% of our patients received nocturnal supplemental oxygen after titration in contrast to only 15% in Priou’s series (this rate was not specified in Budweisser’s study). Supplemental oxygen therapy (SOT) was the only independent predictor of mortality in Priou’s report, but the patients who received it were sicker at baseline and the study was limited by its observational design. The use of SOT to prevent nocturnal oxygen desaturation is still an open question, although this therapy failed to demonstrate a survival advantage in other pathologies like COPD [26]. On the other hand, given the high prevalence of heart disease in OHS patients, the controversial effects of supplemental oxygen on cardiac function must be taken into account. It has been published that oxygen administration can reduce cardiac output [27] in patients with chronic heart failure, but the opposite was also reported [28]. In any case, adding oxygen to NIV was not associated with mortality in our study and the potential role of this therapy in OHS should be prospectively investigated.

We found that clinical history of diabetes, baseline diurnal SaO_2_ < 83%, EPAP < 7 cmH2O after titration and adherence to NIV < 4 hours independently predicted mortality. Given the fact that the majority of death cases were attributable to a fatal cardiovascular event, it should not be surprising that risk factors for cardiac disease—such as diabetes- could predict mortality. It also seems obvious that good adherence to NIV can improve prognosis, as other previous reports have shown [9, 17]. However, since very few patients died during NIV implementation, the association between low baseline diurnal SaO_2_ and mortality is far from clear. A theoretical possibility is that low SaO_2_ values could reflect an underlying severe cardiac disorder, but we don’t have enough data to affirm it. Surprisingly, mortality was neither associated with arterial blood gas values nor with parameters related to respiratory events and gas exchange alterations during sleep, remarking again the effectiveness of nocturnal NIV. The reasons why high EPAP could improve prognosis in OHS patients remain speculative, since data about haemodynamic effects of NIV in this setting are unavailable. Treatment with positive end-expiratory pressure (PEEP) in patients with chronic heart failure is still under debate due to potentially deleterious effect on cardiac output (CO). Early investigations in animals found that PEEP exerted unfavourable haemodynamic effects [29]. However, the theoretical concerns were not reproduced in the clinical setting and a number of mechanisms for the haemodynamic improvement observed with PEEP in patients with heart failure have been described [30]. Anyway, the effects of high ventilatory pressures in OHS haemodynamics should be prospectively investigated in this population of patients.

Although the study is limited by sample size, it should be considered that we only included severe OHS patients. The resulting sample has the advantage of being homogeneous and relatively large. Another limitation of this study is its observational nature. Although we tried to avoid confounding factors by matching the 2 cohorts, a prospective study is preferred to remove them all. In addition, data may be missing, although all the patients were managed by the same medical staff, employing a homogeneous algorithm and, in consequence, very few patients were excluded because lack of fundamental data. Moreover, our results are consistent with available research.

In conclusion, our data demonstrate that the mortality of severe OHS patients is high and substantially worse that of OSAS subjects, even when properly managed and treated from a respiratory perspective. Only few items were able to predict mortality in OHS, namely clinical history of diabetes, baseline diurnal SaO_2_ < 83%, EPAP < 7 cmH2O after titration and adherence to NIV < 4 hours. The protective role of high EPAP should be investigated in a prospective study but, in our opinion, an exhaustive evaluation and treatment of cardiovascular risk factors and cardiac dysfunction is fully warranted in view of our results. These findings contribute to the growing body of knowledge showing that severe OHS should be considered a systemic disease that encompasses respiratory, metabolic and cardiovascular components that require a multimodal therapeutic approach.
